# Women's Awareness and Associated Factors on Preconception Folic Acid Supplementation in Adet, Northwestern Ethiopia, 2016: Implication of Reproductive Health

**DOI:** 10.1155/2018/4936080

**Published:** 2018-07-17

**Authors:** Yitayal Ayalew Goshu, Tewachew Muche Liyeh, Amare Simegn Ayele, Liknaw Bewket Zeleke, Yohannes Tesfahun Kassie

**Affiliations:** ^1^Department of Midwifery, College of Health Sciences, Debre Tabor University, Debre Tabor, Ethiopia; ^2^Debre Markos University, Debre Markos, Ethiopia; ^3^Debre Tabor General Hospital, Debre Tabor, Ethiopia

## Abstract

**Introduction:**

Preconception folic acid supplementation is the provision of folate for reproductive age group women who have a plan to be pregnant. According to different studies, in African countries, there is poor utilization of preconception folic supplementation. So this study aimed at assessing women's awareness on preconception care and its associated factors in Adet, Northwestern Ethiopia.

**Methods:**

A community-based cross-sectional study was conducted from March 1 to April 1, 2016, among 422 reproductive age group women. Systematic random sampling was used to get the study unit, and the data were collected using pretested structured questionnaire via face-to-face interview. The collected data were entered, cleaned, checked using EpiData version 3.1, and finally analyzed using SPSS version 20. Descriptive summary of the data was presented in terms of percentage and frequency. Binary and multiple logistic regressions were used in order to identify predictors using odds ratio at 95% confidence interval.

**Result:**

In this study, a total of 422 reproductive age group women participated with a response rate of 100%. Of a total of 422 participants, 67 (15.9%) of the women had good awareness on preconception folic acid supplementation. Women's awareness on preconception folic acid supplementation was affected by having a chronic health problem, monthly household income, educational status, and a history of family planning use. Women who were educated (AOR 4.77, CI 1.85–6.98), had a history of family planning use (AOR 3.89, CI 1.77–8.55), had a chronic health problem (AOR 3.47, CI 2.68–5.98), and had a better monthly income (AOR 2.6, CI 2.05–6.76) had good awareness than their counterparts.

**Conclusion and Recommendation:**

This study concluded that women's awareness on preconception folic acid supplementation was low. This finding suggests that there is a need to give emphasis and deliver health education about preconception folic acid supplementation for women.

## 1. Introduction

Supplementing folic acid before pregnancy is the provision of folate for reproductive age group women who have a plan to be pregnant, so that the health of women before pregnancy can be promoted and pregnancy-related outcomes can be improved [1, 2]. Folic acid is an important substance which is grouped under essential vitamin B that provides one-carbon molecules for deoxyribonucleic acid (DNA) synthesis, protein synthesis, and methylation of DNA and proteins [3]. A study, which was done to see the association between maternal folic acid intake and the risk of neural tube defects, revealed that the pathway of folic acid plays a critical role in cellular function and human development [1].

According to different interventional studies done in different countries, administering of 0.4 mg of folic acid for the reproductive age group three months before pregnancy, during pregnancy period, and up to one month after pregnancy has been associated with up to 80% reduction in specific congenital anomalies including neural tube defects (NTDs) with associated hydrocephalus, oral facial clefts with or without cleft palate, congenital heart disease, urinary tract anomalies, and limb defects, as well as some pediatric cancers [4–11]. Many articles revealed that preconception folic acid supplementation is associated with increased fetal growth resulting in higher placental and birth weight, and decreased risks of low birth weight and small for gestational age [12–14]. One interventional study also revealed that the consumption of folic acid before conception can decrease the risk of developing anemia and peripheral neuropathy [15]. Despite wide availability of its natural food sources (green leafy vegetables, bananas, and legumes), folic acid deficiency among women of reproductive age is common worldwide, usually as a result of low dietary intake [16, 17].

Articles done in different areas revealed that women's knowledge on the role of folic acid in preventing NTDs as well as the utilization of preconception folic acid is very low [18–20]. But the prevalence of congenitally anomalies, especially neural tube defects, is a major problem worldwide. Moreover, the incidence and prevalence of neural tube defects in developing countries, including African countries, have been reported to be up to fourfold higher than those in developed ones. Different articles show that the lifelong medical and socioeconomic consequences of NTDs in affected children are well known to be worse in low-resource settings [13, 21, 22]. Studies done in developed countries such as Canada, Lebanon, and the Netherlands figured out that knowledge on preconception folic acid supplementation was 81.0%, 71.9%, 53.3%, and 36.8%, respectively [23–26]. But based on different studies done in developing countries such as Nigeria, Jordan, and Pakistan, women's knowledge on preconception folic acid supplementation was 25.5%, 21.2%, and 6.7%, respectively. Moreover, the awareness of women on preconception folic acid supplementation in Ethiopia has not been studied yet [27–29].

Different articles showed that women's awareness on preconception folic acid supplementation is affected by sociodemographic characteristics (such as age, gender, educational status, ethnicity, income, and marital status) and women's reproductive history (history of pregnancy, history of family planning use, health condition, history of ANC visit, parity, and gravidity) [23–29].

Therefore, this study aimed at assessing women's awareness on preconception folic acid supplementation and associated factors in Adet, Northwestern Ethiopia, which is helpful for policy makers to allocate resources and intervene utilization and determinant factors of preconception care for improving of maternal and child health.

## 2. Methods

### 2.1. Setting

This community-based cross-sectional study was conducted in Adet town, which is an administrative town of Yilmana Densa Woreda and located in West Gojjam Zone, Amhara Regional State, from March 01 to 30, 2016. Adet is located 524 kilometers away from Addis Ababa (the capital city of Ethiopia) and 42 kilometers far from Bahir Dar (the capital city of Amhara Regional State) with an altitude of 2, 216 meters above the sea level. According to figures from the Central Statistical Agency in 2015, the estimated total population of the town is 42, 983. Out of this, 21, 234 (49.4%) were men and 21, 749 (50.6%) were women. In this turn, the total number of women who were in the reproductive age group (15–49 years) were 14, 248, which accounts for 33.1% of the total population. The town has three kebeles (the smallest unit of the Woreda) and 13,515 households [30].

### 2.2. Participants

#### 2.2.1. Source Population

All women who lived in Adet were the source of population.

#### 2.2.2. Study Population

All reproductive age group women who lived in Adet were our study population.

#### 2.2.3. Study Unit

Individual reproductive age group woman was our study unit.

### 2.3. Eligibility Criteria

All reproductive age group women who lived in Adet for 6 months and above were included.

### 2.4. Sample Size Determination and Sampling Procedure

The sample size was calculated by using a single-population proportion formula = *Z*^2^*p*  (*p* − 1)/*d*^2^, with assumptions of 50% the population proportion of women's awareness on preconception folic acid supplementation, 95% confidence interval, marginal error of 5% (0.05), and 10% nonresponse rate. All the three kebeles (the smallest unit of the district) were included for study. To reach the study unit, systematic sampling technique was used in all the three kebeles. The first house was selected randomly in one place and every 32nd house for all kebele was asked. The sampling interval of the households in each kebele was determined by dividing the total number of households in the specific kebele by the allocated sample size. When there was no woman in the reproductive age group in the selected house, the nearby house was asked. In case of more than one eligible woman encountered in the selected household, a lottery method was used to determine which woman would be interviewed.

### 2.5. Data Collection Tools and Techniques

An interviewer-administered questionnaire was developed for the purpose of data collection after reviewing relevant literature. It was prepared originally in English and was translated into local language, Amharic, for the purpose of data collection, and then it was translated back to English again for consistency and accuracy by language experts. The questionnaire had sociodemographic parts, reproductive characteristics, and questions related to preconception folic acid supplementation awareness. Face-to-face interview was carried out by three diploma nurses under the supervision of two degree-holding nurses and principal investigator for a period of one month. Training of data collectors and supervisors about purpose of study, handling of ethical issues, and data collection procedures was given for two days. In addition to this, the quality of data was ensured through pretests of 22 women, manual checkup for completeness and accuracy on a daily basis, and proper feedback by supervisor.

### 2.6. Operational Definition

- *Smoking status*: had a history of smoking or currently smokes regardless of amount.
- *Alcohol consumption*: intake of alcoholic drinks on days other than holidays and culturally special ceremony days.
- *Awareness*
  - *Good awareness*: a woman was considered as having good awareness if she had heard about preconception folic acid supplementation and had told the appropriate time of initiation.

### 2.7. Data Analysis

First, the collected data were entered, cleaned, and checked by the software EpiData version 3.1 and then exported to SPSS version 20 for analysis. Descriptive summary of different variables was presented in terms of frequency and percentage by using table and graph. Binary and multivariate logistic regressions were computed to identify predictor variables with an odds ratio at 95% confidence interval. In binary logistic regression, variables which had a *P* value of < 0.25 were transferred into multivariate logistic regression, and finally, variables which had *P* value < 0.05 in multivariate logistic regressions were considered as significantly associated with women's awareness on preconception folic acid supplementation.

### 2.8. Ethical Considerations

Ethical clearance was obtained from the Institutional Review Board of the College of Health Sciences, Mekelle University, and the official cooperation letter was obtained from the Amhara Regional Health Bureau. Finally, the ethical clearance of this study was granted by official cooperation letter of Yilmana Densa Woreda Health Office. Participants consented to take part in the study by signing the informed consent form after they had been thoroughly briefed about the purpose of the study. Participation was on a voluntary basis after written consent, and the responses were kept confidential. The interview was carried out privately in a separate area and confidentiality was ensured through coding.

## 3. Result

### 3.1. Sociodemographic Characteristics

This study was carried out among a total of 422 reproductive age group women with a response rate of 100%. Almost all of the participants (99.8%) were Amhara in ethnicity, and most of the participants (84.8%) were Orthodox Christian in religion. The median age of the participants was 25 years with an interquartile range of 11 years. 35.3% of the respondents had a monthly household income of 100 US dollar or less, and 31.0% of the respondents had high school education. More than half of the participants (59.5%) were married and 26.8% of women were housewives (Table 1).

### 3.2. Reproductive Characteristics

Among a total of 422 participants, 268 (63.5%) have been pregnant before. Among those who have been pregnant before, 6 (2.2%) of the participants had a history of preterm birth and only 5 (1.9%) of the respondents had ever been given birth to a baby with congenital anomaly. Based on the respondents' parity, 154 (36.4%), 53(12.6%), 191 (45.3%), and 24 (5.7%) of the participants were nulliparous, primiparous, multiparous, and grand multiparous, respectively. The majority (58.1%) of the respondents had a history of family planning use, and around 8.3% of respondents had chronic health problems (Table 2).

### 3.3. Preconception Folic Acid Supplementation Awareness

**Figure 1:**
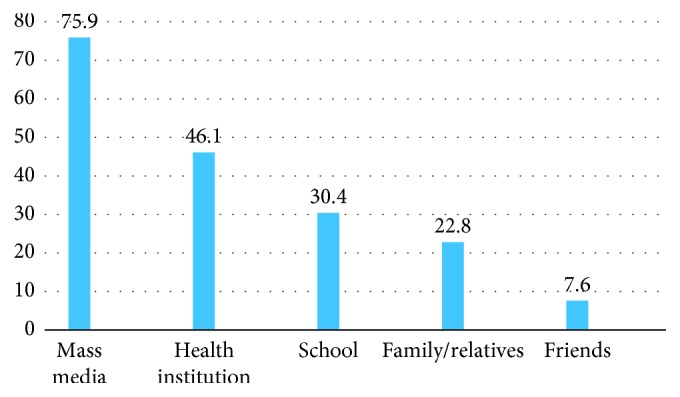
Women's source of information for preconception folic acid supplementation in Adet, Gojjam, Northwestern Ethiopia, 2016 (*n* = 79). Note that the total summation of percentage is more than 100% due to multiple answers.

**Figure 2:**
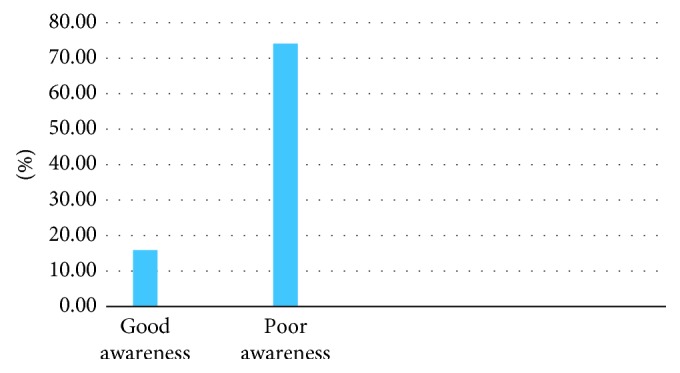
Distribution of women's overall awareness on preconception folic acid supplementation in Adet, Gojjam, Northwestern Ethiopia, 2016 (*n* = 422).

The finding of this study revealed that from a total of 422 participants, only 79 (18.7%) of women have heard before about preconception folic acid supplementation. From those who have heard, the majority of the participants (60 (75.9%)) have heard from mass media and minority (6 (7.6%)) of them have heard from friends (Figure 1). Based on participants' overall awareness on preconception folic acid supplementation, only 67 (15.9%) participants had good awareness (Figure 2). From those who have heard about preconception folic acid supplementation, 67 (15.9%) participants have correctly mentioned the right time of initiation for preconception folic acid supplementation (Table 3).

### 3.4. Associated Factors

In order to check the association between dependent variable and independent variables, first 15 variables were entered into the binary logistic regression. Then, six variables (women's age, educational status, monthly income, a history of family planning use, having a chronic health problem, and a history of pregnancy) with a *P* value of < 0.25 were entered into multivariate logistic regression. Finally, in multivariate logistic regression, only four variables (women's history of family planning use, household monthly income, having a chronic health problem, and educational status) were statically significant (*P* value < 0.05) at 95% confidence interval with women's awareness on preconception folic acid supplementation (Table 4). Women who had primary education were approximately four times more likely to have had good awareness on preconception folic acid supplementation than women who had no formal education (AOR 3.94, CI 2.56–4.78). Women who had a chronic health problem were more than three times more likely to have had good awareness on folic acid supplementation than women who did not have a chronic health problem (AOR 3.47, CI 2.68–5.98).

## 4. Discussion

Folic acid supplementation before pregnancy and just during postpartum period for at least four weeks helps to protect the occurrence of neural tube defects and other congenital abnormalities [8–11]. And having awareness about preconception folic acid supplementation is very important for women to start taking folic acid early.

The findings of this study revealed that sixty-seven (15.9%) participants had good awareness on preconception folic acid supplementation. The result of this study is lower than those of many studies conducted in developed countries like Canada (81%) [23], Lebanon (71.9%) [24], the Netherlands (57.3%) [25], China (34.6%) [29], and Nigeria (25.5%) [28]. This difference may be due to the study population difference, in which previous studies were done among pregnant women, whereas this study was carried out in all reproductive age group women. In addition to this, the lowest awareness in current study may be due to low media coverage in Ethiopia and most of the participants had no higher level education. However, the result of this study is slightly higher than those of studies done in Nigeria (7.4%) and Pakistan (6.7%) [27]. This slight difference of women's awareness with regard to preconception folic acid supplementation is probably due to time variation between the current study and previous studies.

As Table 4 shows, women's educational status, monthly household income, having a chronic health problem, and a history of family planning use were significantly associated with women's awareness on preconception folic acid supplementation in both binary and multivariable logistic regression analysis. Women who had formal education, who had a chronic health problem, whose monthly household income was greater than one hundred US dollar, and who had a history of family planning use had good awareness on preconception folic acid supplementation.

Women's educational status was strongly associated with women's awareness on preconception folic acid supplementation. Women who had primary school education, secondary school education, and college and above were 3.9, 4, and 4.8 times more likely had good awareness on preconception folic acid supplementation than women who had no formal education, respectively (AOR 3.94, CI 2.56–4.78; AOR 4.05, CI 2.79–6.94; and AOR 4.77, CI 1.85–6.98). This finding agrees with studies done in Nigeria, Lebanon, the Netherlands, China, and Pakistan [24, 25, 27–29]. The association may be explained by the fact that when individuals are being educated, they can easily read and understand information regarding folic acid and educated women might have interest to read, listen, and watch any information sources. In addition to this, even information related to folic acid supplementation may be discussed in school, so women who have formal education may have the chance to be aware.

The other important predictors of the current study were monthly household income (AOR 2.62, CI 2.05–7.76), having a chronic health problem (AOR 3.47, CI 2.68–5.98), and a history of family planning use (AOR 3.89, CI 1.77–8.55). The finding of this study is in line with studies done in Lebanon [24], the Netherlands [25], China [29], and France [31]. This is probably due to the fact that women who have a high income may have communication media and they may access information regarding folic acid. The association of having a chronic health problem and preconception folic acid awareness may be explained by the fact that women who had a chronic health problem may seek information about preconception health and pregnancy. Since pregnancy counseling, including preconception care, is given in the family planning unit, women who have used family planning methods might have some sort of information regarding preconception folic acid supplementation.

This study has different strengths. One of the strengths is that as it is community based, it is a representation of the true population. The other strength is the maximum sample size considered. But it has not been ended without limitation; one of the limitations of face-to-face interview is interviewer bias, and it might occur to some extent. Literatures relating to this specific title in African countries were very limited; especially, there was no literature which was done in Ethiopia regarding preconception folic acid consumption. Because of this problem, there was a difficulty in discussion part. As a quantitative study is not good to obtain better in-depth information what the participants have and feel, in-depth information was not studied.

## 5. Conclusion

The finding of this study confirmed that women's awareness on preconception folic acid supplementation is low. The finding of the current study identified that women's educational status, monthly household income, having a chronic health problem, and a history of family planning use were the predictors of women's awareness on preconception folic acid supplementation.

## Figures and Tables

**Table 1:** Sociodemographic characteristics of women in Adet, Gojjam, Northwestern Ethiopia, 2016 (*n* = 422).

Characteristics	Frequency (*N*)	Percent (%)
Age		
15–24	196	46.4
25–34	136	32.2
35–49	90	21.3

Ethnicity		
Amhara	421	99.8
Shinasha	1	0.2

Religion		
Orthodox	358	84.8
Muslim	60	14.2
Protestant	4	1.0

Occupation		
Housewife	113	26.8
Government employee	79	18.7
Market trade vendor	107	25.4
Daily laborer	28	6.6
Student	95	22.5

Educational status		
No formal education	99	23.5
Primary school	97	23.0
Secondary school	131	31.0
College and above	95	22.5

Monthly income		
50$ or less	149	35.3
51–100$	96	22.7
101–150$	55	13.1
151–200$	65	15.4
More than 200$	57	13.5

Marital status		
Married	251	59.5
Divorced	35	8.3
Widowed	17	4.0
Single	119	28.2

**Table 2:** Reproductive characteristics of women in Adet, Gojjam, Northwestern Ethiopia, 2016 (*n* = 422).

Variables	Frequency (*N*)	Percent (%)
Ever been pregnant?		
Yes	268	63.5
No	154	36.5
Total	422	100

History of preterm birth (*n* = 268)		
Yes	6	2.2
No	262	97.8
Total	268	100

History of congenital anomaly (*n* = 268)		
Yes	5	1.9
No	263	98.1
Total	268	100

Counseled about subsequent pregnancy (*n* = 5)		
Yes	2	40.0
No	3	60.0
Total	5	100

Number of alive children (*n* = 268)		
≤2	168	62.7
>2	100	37.3
Total	268	100

Family planning use		
Yes	245	58.1
No	177	41.9
Total	422	100

Having chronic health disease?		
Yes	35	8.3
No	387	91.7
Total	422	100

Types of health problems (*n* = 35)		
HIV/AIDS	7	20
Hypertension	6	17.2
Infertility	5	14.3
Diabetes mellitus	4	11.4
Epilepsy	4	11.4
Renal problem	4	11.4
Asthma	3	8.6
Others	2	5.7
Total	35	100

Others = liver disease and anemia.

**Table 3:** Distribution of women's awareness on the benefit and time of initiation for on preconception folic acid supplementation in Adet, Gojjam, Northwestern Ethiopia, 2016 (*n* = 422).

Characteristics	Frequency (*N*)	Percentage (%)
Time of initiation		
Three months before pregnancy	67	15.9
One month before pregnancy	6	1.4
Two weeks before pregnancy	4	1.0
One weeks before pregnancy	2	0.4
Do not know	343	81.3
Total	422	100

Benefits of preconception folic acid supplementation		
Prevent anemia	35	8.2
Prevent congenital anomaly	18	4.4
Prevent obstructed labor	15	3.5
Prevent weight loss	11	2.6
Do not know	343	81.3
Total	422	100.0

**Table 4:** Factors associated with women's awareness on preconception folic acid supplementation in Adet, Gojjam, Northwestern Ethiopia, 2016 (*n* = 422).

Variables	Awareness		Crude odds ratio (95% CI)	Adjusted odds ratio (95% CI)
	No	Yes		
Educational status				
No formal education	90 (90.9%)	9 (9.1%)	1	1
Primary school	82 (84.5%)	15 (15.5%)	1.83 (1.096–4.813)^*∗∗*^	3.94 (2.56–4.78)^*∗∗*^
Secondary school	109 (83.2%)	22 (16.8%)	2.02 (1.247–5.039)^*∗∗*^	4.05 (2.79–6.94)^*∗∗*^
College and above	74 (77.9%)	21 (22.1%)	2.84 (2.577–10.633)^*∗∗*^	4.77 (1.85–6.98)^*∗∗*^

Monthly income				
50$ or less	132 (88.6%)	17 (11.4%)	1	1
51–100$	85 (88.5%)	11 (11.5%)	1.01 (.546–1.852)	1.26 (.3–1.67)
101–150$	42 (76.4%)	13 (23.6%)	2.40 (1.074–4.062)^*∗∗*^	2.62 (2.05–6.76)^*∗∗*^
151–200$	52 (80.0%)	13 (20.0%)	1.94 (.847–3.078)^*∗*^	1.04 (.38–2.80)
More than 200$	44 (77.2%)	13 (22.8%)	2.29 (.793–3.072)^*∗*^	1.50 (.35–3.23)

Health problem				
Yes	30 (85.7%)	5 (14.3%)	1. 15 (.792–3. 353)^*∗*^	3.47 (2.68–5.98)^*∗∗*^
No	35 (73.4%	62 (16.0%)	1	1

History of family planning use				
Yes	189 (77.3%)	56 (22.7)	4.47 (3.82–6.84)^*∗∗*^	3.89 (1.77–8.55)^*∗∗*^
No	166 (93.9%)	11 (6.1%)	1	1

^*∗∗*^ *P* value < 0.05; CI = confidence interval.

## Data Availability

The datasets generated during the current study are available from the corresponding author based on reasonable request via email and phone call.

## Abbreviations

AOR: Adjusted odds ratio
CI: Confidence interval
NTDs: Neural tube defects.
